# Using early on-treatment circulating tumor DNA measurements as response assessment in metastatic castration resistant prostate cancer

**DOI:** 10.18632/oncotarget.28599

**Published:** 2024-07-02

**Authors:** S.H. Tolmeijer, E. Boerrigter, N.P. Van Erp, Niven Mehra

**Keywords:** ctDNA, prostate cancer, liquid biopsy, biomarker, androgen receptor pathway inhibitors

Metastatic castration resistant prostate cancer (mCRPC) is lethal, but the number of life-prolonging systemic treatments available for mCRPC has expanded over the years [1]. Real world data suggest that the most common first-line therapy for mCRPC was treatment with an androgen receptor pathway inhibitor (ARPI), being either enzalutamide or abiraterone [2–4], although more patients will nowadays receive ARPI and/or docetaxel already for hormone sensitive prostate cancer (HSPC) [5–10]. Recent clinical trial data suggest potential benefit of adding poly-ADP ribose polymerase inhibitors (PARPi) or lutetium-117-prostate-specific membrane antigen (LuPSMA) to first-line mCRPC treatment with ARPIs in a subset of patients [11, 12]. As these different drug classes are associated with different toxicity profiles and significant costs, it is highly important to identify which patients experience durable benefit from monotherapy ARPI and which patients would potentially benefit from treatment intensification or therapy switch.

Research by Tolmeijer et al. 2023, published in Clinical Cancer Research [13], suggests that the detection of circulating tumor DNA (ctDNA) at baseline and 4-weeks after treatment initiation can predict response durability to first-line ARPIs. In two prospective observational multicenter studies, the researchers examined the ctDNA fraction (the proportion of total plasma cell-free DNA that is tumor-derived) in 81 patients with mCRPC before start of therapy and 4-weeks after treatment initiation. Baseline ctDNA was detected (≥1% ctDNA) in 59% of patients and the detection had strong prognostic implications, consistent with literature [14–16]. Importantly, persistent ctDNA detection 4-weeks after treatment initiation was associated with a 4.8 times shorter progression free survival (PFS) and 5.5 times shorter overall survival (OS) compared to patients with undetected ctDNA at baseline and 4-weeks. In contrast, patients converting from detected to undetected ctDNA within 4-weeks of treatment had a similar PFS compared to patients with undetected ctDNA at both timepoints. Importantly, the OS for patients with detected to undetected ctDNA was longer compared to patients with persistent ctDNA detection (28 vs. 16 months). Thus, early changes in ctDNA during treatment could be informative to predict treatment outcomes.

The authors were able to identify patients with a non-durable response (PFS <6 months) with high accuracy using 4-weeks on-treatment ctDNA. Early identification of non-durable responses enables a window of opportunity for treatment intensification for those with insufficient disease control, while sparing additional toxicity for patients with a deep and durable response on ARPI monotherapy. Current clinical tools (e.g., PSA measurements) do not allow accurate prediction of response before radiographic imaging at 3 and/or 6 months [17]. The data of Tolmeijer et al. 2023 suggests that 94% of patients with a durable response had undetected ctDNA at by 4-weeks of treatment, while 85% of patients with a non-durable response had persistent ctDNA detection at 4-weeks. Importantly, ctDNA detection outperformed PSA response at both 4 and 12 weeks for the prediction of non-durable response, with a significant proportion of patients still reaching a PSA50 at 4-weeks (38%) and 12 weeks (27%) despite early radiographic progression. Interestingly, changes in ctDNA from detected to undetected during treatment seemed to be associated with deep PSA responses, with 89% of patients achieving a PSA50 response by 12 weeks. Although the combination of using PSA responses and ctDNA changes could be further investigated, persistent ctDNA detection alone at 4-weeks had a positive predictive value (PPV) of 88% and negative predictive value (NPV) of 92% for identifying non-durable response and could be considered as a standalone tool to nominate patients for treatment intensification or switch by 4-weeks.

Several questions remain for the optimal utility of on-treatment ctDNA to guide patient management. First, the 4-weeks on-treatment timepoint selected by Tolmeijer et al. was pre-specified in their protocol and is practical in clinical practice for most ARPIs, but the utility of using even an earlier timepoint could be investigated. When selecting earlier timepoints the pharmacology of the investigated drugs need to be considered. Some of the APRIs reach steady-state concentrations after just a few days (abiraterone, darolutamide), while others research steady-state after multiple weeks to a month (apalutamide, enzalutamide) [18]. Confirmation of the first on-treatment ctDNA measurement with a second timepoint could improve accuracy, as suggested in other cancer types [19, 20], but might also lead to delayed interventions. Additionally, there are different assays to assess ctDNA fraction in prostate cancer all with different limits of detections. The custom assay used by Tolmeijer et al. has been shown to have strong prognostic utility for baseline ctDNA assessments [14–16]. The limit of detection of this assay is approximately 1%, which is in line with most other commercially available assays [21]. Use of personalized mutation panels could boost the limit of detection to below 1% and potentially refine outcome prediction. However, these assays are challenged by the requirement of representative tissue samples, which can be difficult to obtain for mCRPC [22–24]. It is also unknown whether very low on-treatment ctDNA levels are clinically meaningful in metastatic disease. Finally, there is a need to further investigate the ctDNA changes in the context of different disease stages and different therapeutic agents, such as taxane chemotherapy or PARPi [25–27], to assess the broader applicability of on-treatment ctDNA measurements. Eventually, a prospective clinical trial testing ctDNA guided therapy versus current clinical practice would be warrant to assess the potency of on-treatment ctDNA for therapy guidance.

When designing a prospective randomized-controlled interventional trial with ctDNA guidance in the experimental arm, several crucial considerations are based on the accuracy of the ctDNA test. If the ctDNA test has a sufficiently high PPV/NPV to detect non-durable responders, acknowledged and accepted by both physicians and patients, an early treatment switch in the experimental arm can be possible. This may be achieved by implementing a second on-treatment ctDNA measurement boosting confidence in the ctDNA results, especially since the abundance of total cell-free DNA can be influenced by multiple biological processes (inflammation, exercise, etc.,) [28–30] and ctDNA detection can be impacted by technical variability [20]. To cause limited delay, the ctDNA sampling could be repeated after one week (4 and 5 weeks on-treatment), with patients only being considered for treatment switch when both timepoints have detected ctDNA. This dual measurement strategy has previously prospectively been used for ctDNA-guided adjuvant therapy [31]. As an on-treatment ctDNA-test will unlikely be perfect in identifying non-durable responders, it may be preferable to intensify patients with persistent ctDNA detection on ARPIs with a second life-prolonging agent. Intensification strategies for genomically unselected patients can include addition of a taxane or radioligand, such as cabazitaxel or LuPSMA. Patients with strong PSMA avid lesions may particularly benefit from intensification with LuPSMA as recently reported [12]. However, as ctDNA testing provides us with a genomic profile of the tumor, it gives us the opportunity to consider genome matched switching or intensification strategies. Intensification for genomically selected patients may include addition of a PARPi or PI3K-mTOR-AKT inhibitor. Interestingly, in the study of Tolmeijer et al. 6/29 (21%) of patients with persistent ctDNA detection at 4-weeks had a pathogenic BRCA2 alteration, making them suitable candidates for ARPI intensification with PARPi [11]. Furthermore, more rare homologous recombination repair alterations in genes such as BRCA1, PALB2 and CDK12 also appear to be candidates for ARPI-PARPi intensification regimens [32]. Although genomic matched treatment intensification could be beneficial for patients, it will complicate comparison with the control arm of a randomized trial. This could partly be resolved by reporting the additive PFS of ARPI monotherapy (PFS1) and the second agent of physicians choice (PFS2) in the control arm. Other valuable outcome measurements will be comparison of OS, quality of life and cost analysis per arm.

In conclusion, results of Tolmeijer et al. show the promise of on-treatment ctDNA detection as early read out for treatment response to potentially help guide treatment management of patients with mCRPC. We are excited for future research refining and defining necessary considerations for on-treatment ctDNA measurements and are looking forward to prospective validation of the results.
